# Cost-Effectiveness Analysis of a National Neonatal Hearing Screening Program in China: Conditions for the Scale-Up

**DOI:** 10.1371/journal.pone.0051990

**Published:** 2013-01-16

**Authors:** Ruoyan Gai Tobe, Rintaro Mori, Lihui Huang, Lingzhong Xu, Demin Han, Kenji Shibuya

**Affiliations:** 1 School of Public Health, Shandong University, Jinan, Shandong Province, China; 2 Department of Health Policy, National Center for Child Health and Development, Tokyo, Japan; 3 Beijing Tongren Hospital, Beijing, China; 4 Beijing Institute of Otolaryngology, Key Laboratory of Otolaryngology Head and Neck Surgery, Ministry of Education, Beijing, China; 5 China WHO Collaborating Center for the Prevention and Rehabilitation of Hearing Impairment, Beijing, China; 6 Department of Global Health Policy, Graduate School of Medicine, The University of Tokyo, Tokyo, Japan; Johns Hopkins Bloomberg School of Public Health, United States of America

## Abstract

**Background:**

In 2009, the Chinese Ministry of Health recommended scale-up of routine neonatal hearing screening - previously performed primarily only in select urban hospitals - throughout the entire country.

**Methods:**

A decision analytical model for a simulated population of all live births in China was developed to compare the costs and health effects of five mutually exclusive interventions: 1) universal screening using Otoacoustic Emission (OAE) and Automated Auditory Brainstem Response (AABR); 2) universal OAE; 3) targeted OAE and AABR; 4) targeted OAE; and 5) no screening. Disability-Adjusted Life Years (DALYs) were calculated for health effects.

**Results and Discussion:**

Based on the cost-effectiveness and potential health outcomes, the optimal path for scale-up would be to start with targeted OAE and then expand to universal OAE and universal OAE plus AABR. Accessibility of screening, diagnosis, and intervention services significantly affect decision of the options.

**Conclusion:**

In conclusion, to achieve cost-effectiveness and best health outcomes of the NHS program, the accessibility of screening, diagnosis, and intervention services should be expanded to reach a larger population. The results are thus expected to be of particular benefit in terms of the ‘rolling out’ of the national plan.

## Introduction

Globally, hearing impairment is the third leading type of disability [1]. Incidence of permanent congenital and early-onset hearing impairment (PCEHI) is estimated 2–4 infants per 1000 live births [2]. As adequate auditory stimulation in early childhood is fundamental for optimal speech and language development as well as for the acquisition of literacy skills [3], a failure to undertake early hearing detection and intervention (EHDI) within the first year of life for PCEHI can lead to significantly and irreversibly impaired language acquisition, learning and speech development early in life and low educational and occupational performance in adulthood [4]–[8].

The NHS programs have reduced the age of detection of child-onset hearing impairment significantly, and made EDHI possible [9]–[12]. Essentially there are two strategies for NHS: universal screening, covering all live births; and targeted screening, or so-called selective screening, which targets those with one or more risk factors, including gestational age < = 34 weeks, low birthweight (<1500 g), family history, TORCH infections, neurological disorder, hyperbilirubinemia, craniofacial anomalies, syndromes known to associated with hearing loss, and severe birth asphyxia (Apgar <7 at 5 min) [13]. Universal NHS can detect infants with the disorder who have no known risk factors associated with PCEHI, which accounts for approximately 50% of PCEHI cases [12], [14]. The implementation of the screening program has been shifted from the targeted to the universal and achieved relevantly high coverage in developed countries, such as US and UK [15], [16]. The major limitation results from the very high probability of false-positive results due to the low prevalence of PCEHI, which may incur unnecessary referral costs and much parental anxiety [17], [18].

The NHS program in China has been introduced in a few urban hospitals in metropolitan cities since the 1990s with the scale of its implementation gradually expanding, mainly in general hospitals and maternal and child hospitals (MCH) in urban areas. The protocol includes two-stage screening using Otoacoustic Emission (OAE) or OAE plus Automated Auditory Brainstem Response (AABR). As the most common screening technique, the result of OAE reflects the function of cochlea; while it can be influenced by the condition of outer and middle ear, causing the false positive. Moreover, some conditions such as auditory neuropathy and the impairment in cochlear inner cells cannot be detected by OAE, causing the false negative. As with OAE, AABR is an accurate and convenient tool to assess the whole auditory pathway, including the condition of outer, middle, and inner ear. The limitation of AABR is that it isn't sensitive enough to the hearing loss at low and high audio frequency. Previous practices in European countries indicated that the two-stage OAE plus AABR was a likely solution of high false positive because refer rates at time of hospital discharge from such programs were reported to be much lower than those in programs that used just OAE screening [19]. Therefore, the combination of OAE and AABR has been regarded as an optimal practice for the screening, particularly effective to detect acoustic nerve diseases.

In 2009, the Ministry of Health (MOH), China decided to scale-up the NHS program, with a newly launched national plan to integrate the program into the maternal and child health services package as a part of congenital disease screening. The 2-stage OAE plus AABR is recommended as the protocol of the screening. In the first stage neonates are screened within two to seven days after birth before they are discharged from the hospital. Those who fail OAE or both are then referred for a second stage confirmation test within 42 days of birth by OAE or AOE plus AABR in order to ensure that the hearing disorder is diagnosed before six months of age. If the diagnosis is confirmed, fitting of hearing aid or a surgery for cochlear implant will be provided to those children, and then they will be introduced to rehabilitation centers to receive language rehabilitation by specialists before 12 months of age to 6 years, the age when primary school is entered. In the national plan, although universal screening by the two-stage OAE plus AABR is recommended, targeted screening or screening using OAE alone is considered as an alternative option for regions with limited financial and technical capacity. Several questions consequently may arise from the current policy: which strategy (universal or targeted) should be prioritized; and which protocol (OAE or OAE plus AABR) should be adopted? At what level of related services provision should it scale-up the NHS program from a targeted strategy to a universal strategy, considering diversified socioeconomic status of different regions?

Therefore, this study aims to provide support for the decision making of national and provincial policy makers on the implementation of nationwide NHS programs. The objectives are to assess the cost-effectiveness of different NHS strategies, to explore the impact of the accessibility of screening, diagnosis and interventions on cost-effectiveness, and to determine the conditions that are necessary for NHS to be scaled-up in different regions.

## Methods

### Study overview

A mathematical simulation model consisting of neonates in China was developed, in which each cohort will experience the currently observed age-specific mortality rates. Costs and health effects gained from the screening program for the simulated neonates were compared among five strategies: 1) universal screening using OAE and AABR (uni.OAE+AABR), 2) universal screening using OAE (uni.OAE), 3) targeted screening using OAE and AABR (select.OAE+AABR), 4) targeted screening using OAE (select.OAE), and 5) no screening. The major outcomes were the number of disability-adjusted life years (DALYs) averted. The NHS program cannot change the pattern of the incidence in the population, but rather, potentially improve language, cognitive and psychosocial outcomes by EHDI.

### Decision model

**Figure 1:**
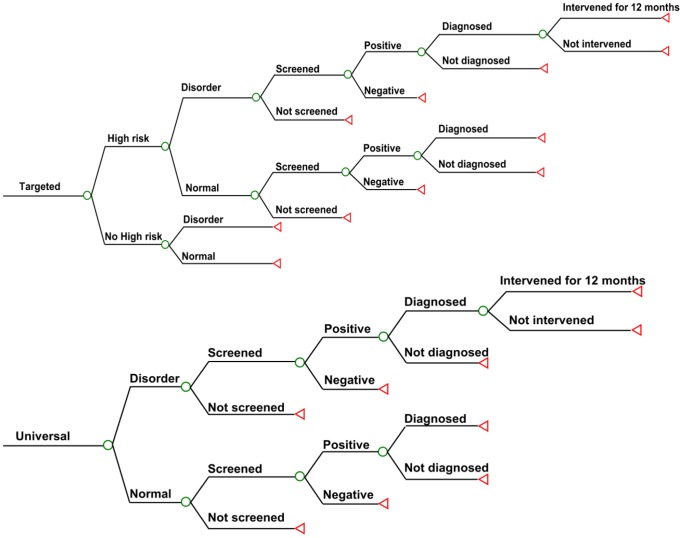
Decision tree for cost-effectiveness analysis of different screening strategies among all simulated live births in China.

A natural history model of infants with and without hearing impairments was developed and the impact of the five strategies as described above was incorporated by using TreeAge Pro 2009 (TreeAge Software Inc., Boston, MA, USA) (**Figure 1**). Incorporating the prevalence and the current practice of NHS in China into its design, this model was used to determine the proportion of infants with PCEHI in the simulated cohort who would potentially benefit from the NHS program, where detection before 6 months of age would occur by a process of screening and diagnosis and with intervention occurring before 12 months of age. The model considered bilateral moderate and severe hearing loss. Unilateral and mild cases were not included in the model, as the disease weight was not available. Costs associated with screening, diagnosis, and interventions were considered. It was assumed that follow-up defaulters from screening, diagnosis and interventions were unable to acquire treated health outcomes and DALYs for those cases cannot be averted.

### Probabilities of health outcomes and sensitivity analysis

The estimates of probability parameters which potentially influence the effect size of different strategies included the proportion of infants with one or more risk factors (based on the criteria of JCIH [13]), the prevalence of PCEHI in the general population, the prevalence of PCEHI in high-risk infants, the sensitivity and specificity of OAE plus AABR, the sensitivity and specificity of OAE, coverage of the screening program, and the diagnosis rate and intervention rate.

Based on the characteristics of the model, one-way sensitivity analysis was performed for uncertainty of these key parameters. Upper and lower boundaries for parameters were changed to evaluate the impact of parameters on the robustness of the model. **Table 1** summarizes the baseline and plausible range for the sensitivity analysis of parameters and costs. The ranges for the sensitivity analysis are derived from the minimum and maximum figures from the empirical and literature-based data. Information on the proportion of infants with one or more risk factors, the prevalence of PCEHI in the general population, the prevalence of PCEHI in high-risk infants, for the sensitivity and specificity of OAE and AABR, and for the sensitivity and specificity of OAE was obtained from literature reviews, as showed in **Table 1**. To explore relationship between coverage of the screening program, and the diagnosis rate and intervention rate, we varied those three parameters in a three-way sensitivity analysis as well.

**Table 1:** Parameter values and plausible ranges for probability variables used in baseline and sensitivity analysis.

	Baseline	Range for sensitivity analysis	References
Live-born neonates	15987609		National census in 2009
urban	7184859		National census in 2009
rural	8802750		National census in 2009
High-risk infants	7%	6–8%	[37]
Prevalence in all live births	0.30%	0.1–0.6%	[38]–[41]
Prevalence in high-risk infants	3.00%	2.5–5%	[42]–[47]
Sensitivity of OAE+AABR	95%	90–100%	[14], [48], [49]
Specificity of OAE+AABR	95%	80–100%	[14], [48], [49]
Sensitivity of OAE	90%	90–100%	[14], [48], [49]
Specificity of OAE	85%	80–100%	[14], [48], [49]
Coverage	40%	15–99%	Pilot data
Diagnosis rate	50%	20–95%	Pilot data
Intervention rate	50%	10–95%	Pilot data

### Cost estimates

We followed the guidelines developed by the World Health Organization (WHO) [20] to estimate costs for the program implementation. All costs were discounted to their net present values at an annual rate of 3%.

Program-level costs included the capital costs of offices (buildings), furniture and equipment; the recurrent costs included such things as the salaries of the personnel, materials and supplies, utilities, equipment maintenance, database management, training and transport. Patient-level costs were composed of the registration fee, screening tests, diagnosis tests, drugs, treatment with a hearing aid, treatment with a cochlear implant, rehabilitation courses, and transportation charges for diagnostic procedures and treatments. The capital investments of buildings, furniture and equipment were annualized by depreciation, for which the life spans were decided by the Ministry of Health and Ministry of Finance, China [21].

In the model, the baseline figures were the average costs of the program implementation combining the program-level and the patient-level per patient for screening, diagnosis, and intervention, and the range from the minimum to the maximum were introduced to sensitivity analysis. Costs were first collected in RMB, the Chinese currency, costs across different years were adjusted with the price level in 2009 based on the GDP deflator, and then transferred into international dollars (Int$), by dividing Purchasing Power Parities [22]. Data for the cost estimates came from the annual financial report of screening facilities, diagnosis centers and rehabilitation facilities in the surveyed provinces, with the cooperation of experts in hospital accounting. Sheet for the data collection and estimated unit cost are attached in **Appendix S1** and **Appendix S2**, respectively.

### Estimates of population health effects and cost-effectiveness

Health effect is expressed as the number of DALYs averted as a result of the screening program [23]. Average cost-effectiveness ratio (ACER) is calculated for each screening strategy by summing total costs and total health effects in terms of DALYs averted. WHO defined interventions with ACER less than three times of the gross domestic product (GDP) per capita as the cost-effective [24]. Incremental cost-effectiveness ratios (ICER) in different screening strategies are calculated by dividing the incremental costs by the incremental health effects, in order to determine the priority to purchasing those services in different budget levels.

### Data collection

Data collection in the field included two components: costs related to the screening program, which were acquired from general hospitals and MCH providing screening and diagnosis services, and rehabilitation facilities; and transition probability parameters including coverage of the screening program, diagnosis rate and intervention rate, acquired from the regional database.

A field survey to collect data in six provinces: Beijing, Shandong, Hebei, Henan, Jiangxi, and Guangxi, considering geographical and socioeconomic representativeness in China. In all surveyed six provinces, the database has been established at the provincial and district level for monitoring and evaluation of the NHS program. Data needed to calculate transition probability parameters were duplicated from the database. For details please refer to **Appendix S1**.

## Results

### Cost-effectiveness of different screening strategies

In the simulated synthetic cohort of Chinese live births, the prevalence of PCEHI was estimated as 3 per 1,000 in the whole population. Based on the prevalence and the current practice of NHS in China, our model predicted the proportion of infants with PCEHI in the simulated cohort detected before 6 months of age by the process of screening and diagnosis and intervened before 12 months of age (**Table 2**), and consequently, the costs, health outcomes and cost-effectiveness ratio of different screening strategies. For a program that screens 15,787, 609 live births every year, the incremental number of cases detected with universal OAE plus AABR is 9,112; and the incremental number of people finally receiving EHDI is 4,556. **Table 3** summarizes the estimated costs of the program implementation including program and patient aspects, health effects in terms of DALYs averted, ACER, and ICER for each screening strategy in China.

**Table 2:** Estimation of cases detected and cases intervened by different screening strategies.

Interventions	Prevalence in the population	Cases screened	Cases detected	Cases received EHDI
**Uni. OAE+AABR**	47,963	6,395,044	9,113	4,556
**Uni. OAE**		6,395,044	8,633	4,317
**Select. OAE+AABR**		313,357	4,465	2,233
**Select. OAE**		313,357	4,230	2,115

**Table 3:** Costs, health effects, and cost-effectiveness of different screening strategies.

Interventions	Costs (millions)			Effectiveness	Cost-effectiveness (thousands per DALY averted)	
	Patients	Program	Total (95% CI)	DALYs averted (95% CI)	Average (95% CI)	Incremental (95% CI)
**Uni. OAE+AABR**	38	236	274 (163–460)	7,710 (6,910–8,520)	36 (20–63)	163 (90–317)
**Uni. OAE**	44	164	208 (118–306)	7,310 (6,380–8,240)	28 (15–42)	38 (22–58)
**Select. OAE+AABR**	7.5	65	72 (53–98)	3,780 (3,390–4,170)	19 (13–27)	127 (94–180)
**Select. OAE**	7.5	39	47 (33–62)	3,580 (3,130–4,040)	13 (8–17)	13 (8–17)
**No screening**	0	0	0	0	NA	NA

Note: Uni.OAE+AABR = universal strategy using OAE plus AABR; Uni.OAE = universal strategy using OAE; Select.OAE+AABR = targeted strategy using OAE plus AABR; Select.OAE = targeted strategy using OAE.

**Figure 2:**
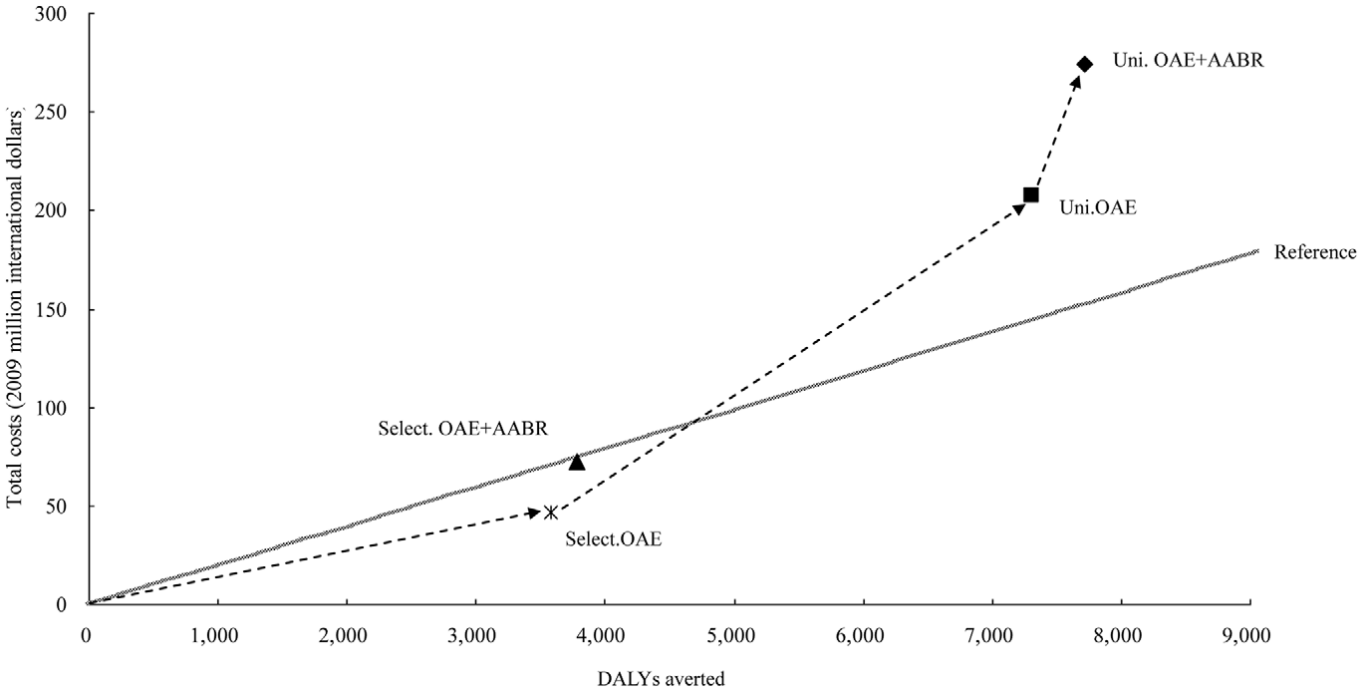
The cost in per Disability-Adjusted Life Years (DALYs) of four screening strategies compared with no screening. Uni.OAE+AABR = universal strategy using OAE plus AABR; Uni.OAE = universal strategy using OAE; Select.OAE+AABR = targeted strategy using OAE plus AABR; Select.OAE = targeted strategy using OAE; Reference = 3 times of GDP per capita (19,700 international dollars).

Based on the WHO's reference, the two targeted strategies remained cost-effective with an ACER of 13,100 (95% CI: 8,400–17,200) and 19,100 (95% CI: 13,300–27,500) international dollars per DALY averted. On the other hand, the universal screening strategies proved to be less cost effective with ACER figures of 28,400 (95% CI: 14,500–41,900) and 35,600 (95% CI: 20,000–63,000) international dollars per DALY averted (**Figure 2**).

Among the four strategies, targeted OAE is most cost-effective. The incremental cost-effectiveness ratio (ICER) suggests that shifting from targeted OAE to targeted OAE plus AABR to universal OAE and to universal OAE plus AABR costs 127,700 (95% CI: 98,000–180,000), 43,000 (95% CI: 25,800–62,400), and 55,000 (95% CI: 32,000–87,000), respectively, for averting per DALY. **Figure 2** showed the optimal path for scale-up, which starts with targeted OAE and then expand to universal OAE and universal OAE plus AABR.

### Sensitivity analyses

The results of sensitivity analysis suggested the variables whose range of uncertainty had a significant impact on the cost-effectiveness of the screening strategies were the program coverage, diagnosis rate, and intervention rate. At the baseline level, by increasing the program coverage up to 85%, universal OAE will be cost-effective. Conditionally, universal OAE plus AABR tend to be cost-effective when diagnosis rate and intervention rate jointly reached from the baseline level to 70%, respectively. Although there was no change on the optimal path for scale-up, with the increase of the three variables, ICER of shifting the strategies gradually reduced.

**Figure 3:**
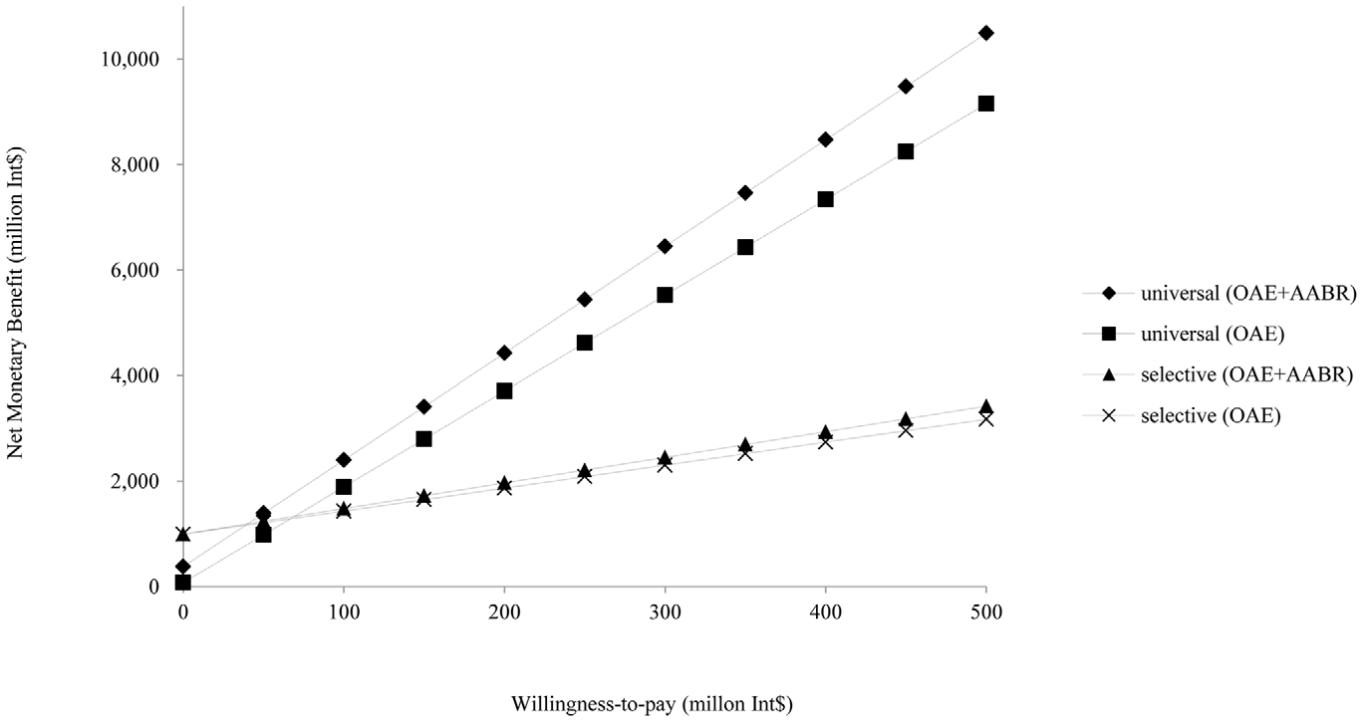
Sensitivity analysis of willingness-to-pay.

**Figure 3** showed the result of sensitivity analysis of willing-to-pay. Based on the data on the effect of the long-term cost saving of the screening program reported elsewhere [25], by increasing willingness-to-pay threshold up to 390 million international dollars, universal OAE plus AABR will achieve much more net monetary benefits compared to other strategies.

**Figure 4:**
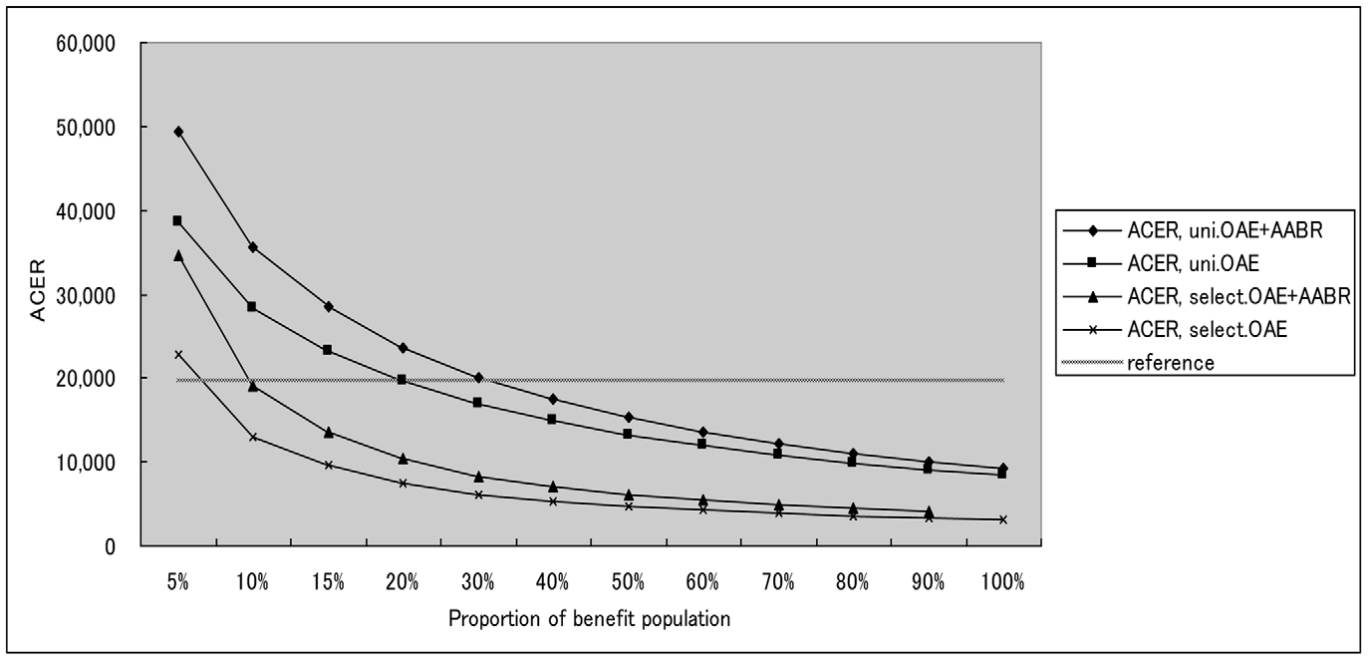
The impact of the benefit ratio on ACER of different screening strategies based on the results of sensitivity analysis. Uni.OAE+AABR = universal strategy using OAE plus AABR; Uni.OAE = universal strategy using OAE; Select.OAE+AABR = targeted strategy using OAE plus AABR; Select.OAE = targeted strategy using OAE; ACER = Average Cost-Effectiveness Ratio.

**Figure 5:**
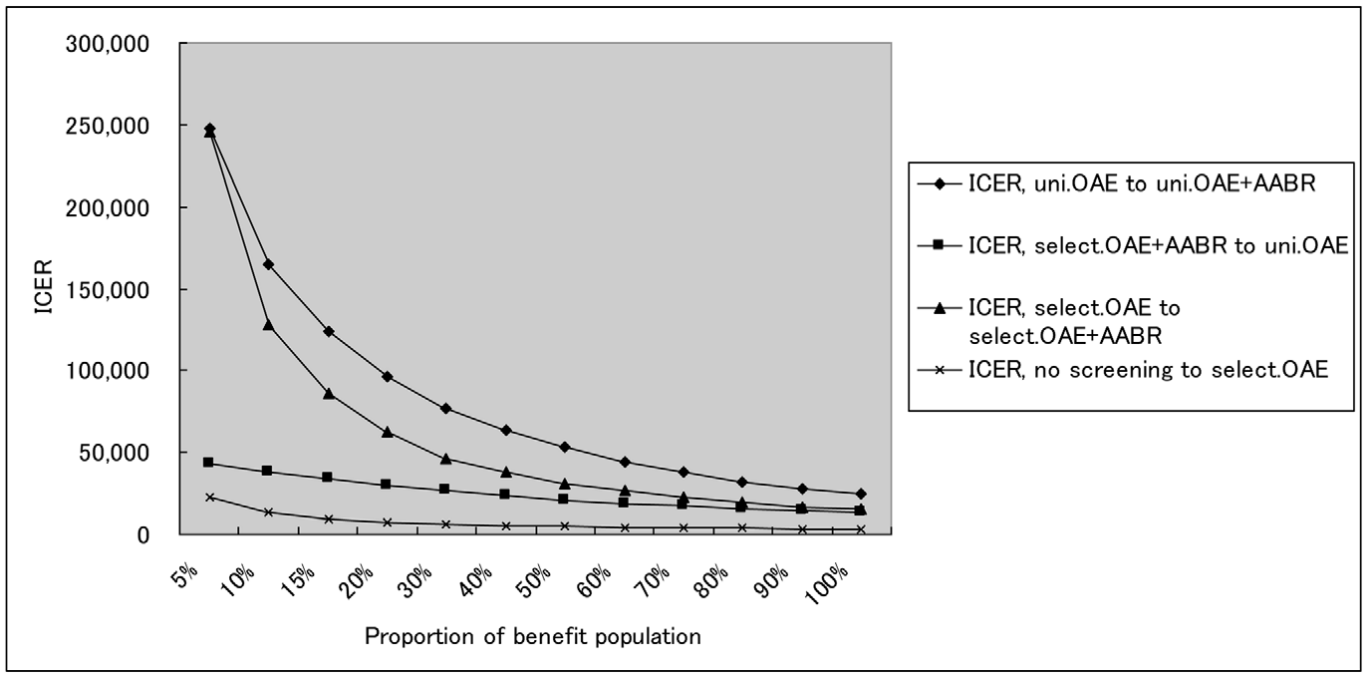
The impact of the benefit ratio on ICER of shifting strategies based on the results of sensitivity analysis. Uni.OAE+AABR = universal strategy using OAE plus AABR; Uni.OAE = universal strategy using OAE; Select.OAE+AABR = targeted strategy using OAE plus AABR; Select.OAE = targeted strategy using OAE; ICER = Incremental Cost-Effectiveness Ratio.

Three-way sensitivity analysis demonstrated that Universal OAE plus AABR gradually tended to be cost-effective when the program coverage, diagnosis rate, and intervention rate jointly varied across their plausible ranges. As shown in **Figure 4** and **Figure 5**, the proportion of the benefit population, which was estimated by multiplying the three variables, reduced ACER and ICER considerably in the four strategies and achieved high cost-effectiveness as it increased. Targeted OAE, universal OAE, and universal OAE plus AABR trended to be cost-effective from the level of 7%, 20% and 30%, respectively.

## Discussion

This is the first study to estimate the costs and health effects of a screening program for neonatal hearing impairment in the developing world. Factors such as limited funding, shortages of manpower, and the inadequate provision of follow-up and support services have prevented the implementation of the NHS program in the vast majority of developing countries [26]. Although the study design used here was similar to those of some previous research approaches in developed countries [27]–[31], this study considered not only direct indicators such as the number of infants diagnosed and who received an intervention but also DALYs averted. The advantage of calculating costs per DALY averted is that it allows a comparison to be made with other interventions and other regions. Such the indicator comprehensively measures the disease burden averted by the screening program at different options and conditions.

The goal of the NHS program in China is to establish a nationwide hospital-based universal program and to continuously expand diagnosis and intervention services [32]. The rationale for implementing a universal strategy is that it can detect more deaf infants, providing a greater opportunity for them to experience normal language development, while also providing overall benefits in terms of the reduction in disability and the improvement in health and well-being of the Chinese population. Conversely, our results at a national glance seem to prove the targeted strategies were more cost-effective than the universal strategies. Does it mean that the MOH's plan is too ambitious to achieve at the moment? The answer depends on a huge geographical gap on the socioeconomic status. The screening program adopted in China is similar to that in developed countries; while compared to the implementation in those developed countries, the coverage of the screening program is diversified by regions with different socioeconomic status and the EHDI rate among the children with PCEHI is much lower and there remains a huge regional diversity in terms of implementation. The decision making in China is more complicated, as policy makers face key issues in the selection of the strategies and the protocols after the launch of the national policy for the scale-up. Therefore, our study provides a reference to different regions rather than a standard guideline after the launch of the national policy to scale up the screening program.

Results of sensitivity analysis suggests that not only the program coverage, but also the accessibility of the consequent diagnosis and intervention services after screening, are key factors for the scale-up of the program. At the regional level, these factors tend to parallel to the diversified regional socioeconomic status. The decision making should depend on the regional conditions: current implementation and accessibility of related services, as well as feasibility based on health resources in financial, human resource and material aspects. The study indicated that the optimal path for the scale-up is targeted OAE, universal OAE and ultimately universal OAE plus AABR. Targeted OAE plus AABR should not be considered due to the expensive ICER, underlying which is a limited beneficial population, caused by the targeted strategy. For the scale-up from the targeted to the universal strategies, the beneficial population needs to be expanded to more than 20%. Therefore, for those regions the current implementation cannot reach such the proportion of the beneficial population, particularly in rural and remote areas, targeted OAE is feasible.

In those regions currently targeted OAE is dominated, public investment to the related services for detection and rehabilitation is crucial to improve the cost-effectiveness of the universal strategies with better health outcomes, particularly universal OAE plus AABR. Compared to screening by OAE alone, the protocol of OAE plus AABR with better sensitivity and specificity saves costs by the false positive and detects more PCEHI cases. ICER falls down and becomes extremely close to universal OAE, as the program coverage and accessibility of detection and rehabilitation services increase, suggesting a good cost-effectiveness of the option. By increasing financial investment, it will avert more disease burden and have better monetary benefits. Therefore, in long-term, Universal OAE plus AABR should be the ultimate goal of the scale-up.

After the launch of the national policy to scale up the screening program, coverage of the screening program has significantly increased with the political commitment. On the other hand, availability of the related services for detection and rehabilitation remain as a “bottleneck” for the scale-up. Medical costs for diagnosis and interventions tend to be catastrophic and need to be fully covered by medical insurance or aid rather than an out-of-pocket payment. According to our pilot studies and the national statistics, the patient costs for diagnosis and intervention services exceeded about 10 times of the annual household expenditure [33]. Moreover, ensuring human resources needs a long-term effort [34]. In China, the audiologist and the specialist in hearing rehabilitation is of severe shortage, constraining the scale-up and the quality of intervention services. Until 2008, the total number of audiologist and specialist in hearing rehabilitation was only about 100, far from the real need of the professional rehabilitation services for about 1,500,000 people with hearing impairment [35].

Our study was restricted by a limited availability of data and evidence. First, although several scientifically sound studies demonstrated the benefits of early detection and intervention on speech and language outcomes, there is no study on the long-term psychological and educational outcomes and consequently the long-term benefits of the program cannot be exactly evaluated. Moreover, in China, there is lack of population-based study to survey the epidemiological situation of PCEHI in the national level. Data on the prevalence used in this study derived from crude estimation based on the number of hearing disable population and our pilot studies for the implementation of the screening program. Last but still important, the disability weight is not specified by severity of hearing impairment and only accounted for adult-onset hearing loss. The prevalence in different severity is not available in China, we cannot take consideration of this factor, which potentially has an impact on language outcomes [5], [36].

In conclusion, to achieve cost-effectiveness and best health outcomes of the NHS program, its benefit population should be expanded by improve the accessibility of screening, diagnosis and intervention services. Depending on the program coverage and the availability of the related services for detection and rehabilitation, the universal strategies would be dominated in the developed provinces where screening, diagnosis and intervention services benefit over 20% of children with the disorder. In other regions, targeted OAE is temporarily more realistic, and related services need to reach 7% of the beneficial population. The regional policy makers should prioritize the investment to the related services for detection and rehabilitation and have an endeavor to improve its accessibility. The reference data from this study are thus expected to be of particular benefit in terms of the ‘rolling out’ of the national plan.

### What is already known on this topic

The neonatal hearing screening program reduced the age of detection of child-onset hearing impairment significantly and made early hearing detection and intervention possible. The universal strategy has good cost-effectiveness in developed countries and has been widely applied.

### What this study adds

In China, the accessibility of screening, diagnosis and intervention services diversified in different regions, leading to different cost-effectiveness of strategies and health effects. To achieve cost-effectiveness and best health effects, its benefit population should be expanded by improve the accessibility of those related health services.

## Supporting Information

Appendix S1 **Information list for data collection.**
(DOCX)

Appendix S2 **Parameter values and plausible ranges for cost estimates per case used in baseline and sensitivity analysis (millions of international dollars).**
(DOCX)
