# Supplementary material for: Cost-Effectiveness Analysis of a National Neonatal Hearing Screening Program in China: Conditions for the Scale-Up
Source: PLoS One. 2013 Jan 16;8(1):e51990. doi: 10.1371/journal.pone.0051990 (PMC3547019; doi:10.1371/journal.pone.0051990)
Supplement: Appendix S1 — Information list for data collection. (DOCX) [file pone.0051990.s001.docx]

**Appendix S1. Information list for data collection**

| **Part I. <For maternal and child health division of the local health agency>** |
| --- |
|  |
| *Basic information* |
| 1. Number of screening facilities in the district: |
| 2. Screening strategy in the district: |
| *universal screening* |
| *selective screening* |
| 3. Methods of screening |
| OAE |
| OAE+AABR |
| 4. Standard price of screening test (first stage + second stage) |
| 5. Number of diagnostic center(s) assigned by the local health agency: |
| 6. Methods of diagnosis tests (please give details) |
|  |
| 7. Standard price of diagnostic tests |
| 8. Referral system between screening facilities and diagnostic center(s) |
| *yes* |
| *no* |
| 9. Number of live-born neonates in 2009 (in the surveyed district) |
| 10. Number of live-born neonates in 2009 (in the located province) |
| 11. Proportion of neonates with high risks |
| 12. Prevalence of neonatal hearing loss |
| (1) in the general population |
| (2) in those with high risk |
| 14. Number of neonates who were screened in 2009 |
| 15. Number of neonates referred after the screening results (in 2009) |
| (1) Referral rate in the first step: |
| (2) Follow-up rate to the second step: |
| (3) Loss rate to the second step: |
| (4) Referral rate in the second step: |
| 16. Sensitivity and specificity of screening techniques |
| (1) Sensitivity |
| (2) Specificity |
| 17. Number of infants diagnosed among those screened |
| (1) diagnosed within 6 months (+) (-) |
| (2) diagnosed after 6 months (+) (-) |
| 18. Number of infants intervened among those diagnosed |
| (1) intervened within 12 months |
| (2) intervened after 12 months |
| 19. Number of follow-ups to high-risk infants |
| 20. Number of infants passed the screening test but detected the disorder during the follow-up |

| **Part II-1. <For screening facilities in the district>** |
| --- |
|  |
| ***Cost for neonatal hearing screening program*** |
| ***<program level cost>*** |
| Labour |
| wage and bonus of medical staff engaged in neonatal screening |
| Fixed capital |
| building space |
| screening machines |
| office and medical equipment |
| Operating and management costs |
| outreach costs |
| follow-up costs |
| occupational education |
| management of database and case records |
| Overhead costs |
| electricity |
| communication fee |
| maintenance |
| Consumables |
| medical supplies |
| office equipment |
| Depreciation |
| depreciation rate of fixed capital |

| **Part II-2. <For diagnostic center(s) and rehabilitation center(s) in the district>** |
| --- |
|  |
| ***Basic Information*** |
| Interventions provided in this facility |
| *pharmaceutical treatment* |
| *surgical treatment* |
| *fitting hearing aid* |
| *fitting cochlear implant* |
| *auditory and linguistic training* |
| *social-family rehabilitation guidance* |
| ***Costs estimates*** |
| ***<Facility side>*** |
| Labour |
| Fixed capital |
| building space |
| machines for auditory tests |
| office and medical equipment |
| Operating and management costs |
| occupational education |
| management of database and case records |
| Overhead costs |
| electricity |
| communication fee |
| maintenance |
| Consumables |
| medical supplies |
| office uses |
| Depreciation |
| depreciation rate of fixed capital |
| ***<Patient side>*** |
| Medical and auditory tests for diagnosis |
| Pharmaceutical costs |
| Surgery |
| Hearing aid / cochlear implant |
| Rehabilitation course (unit cost × duration) |
