# Supplementary material for: Cost-Effectiveness Analysis of a National Neonatal Hearing Screening Program in China: Conditions for the Scale-Up
Source: PLoS One. 2013 Jan 16;8(1):e51990. doi: 10.1371/journal.pone.0051990 (PMC3547019; doi:10.1371/journal.pone.0051990)
Supplement: Appendix S2 — Parameter values and plausible ranges for cost estimates per case used in baseline and sensitivity analysis (millions of international dollars). (DOCX) [file pone.0051990.s002.docx]

**Appendix 2. Parameter values and plausible ranges for cost estimates per case used in baseline and sensitivity analysis (millions of international dollars)**

|  |  | **Baseline** | **Range for sensitivity analysis** | |
| --- | --- | --- | --- | --- |
| **Screening** |  |  |  |  |
| ***Program costs*** |  |  |  |  |
| Capital costs | OAE | 240,813,700 | 170,712,611 | 307,291,537 |
|  | OAE+AABR | 429,951,873 | 322,027,568 | 559,461,039 |
| Recurrent costs  (per patient in average) | OAE | 150.49 | 97.28 | 201.53 |
|  | OAE+AABR | 225.74 | 131.12 | 361.18 |
| ***Patient costs*** |  | 28.75 | 10.45 | 52.27 |
| **Diagnosis** |  |  |  |  |
| ***Program costs*** |  |  |  |  |
| Capital costs |  | 15,950,599 | 11,014,773 | 21,467,600 |
| Recurrent costs  (per patient in average) |  | 238.16 | 130.29 | 358.24 |
| ***Patient costs*** |  | 169.89 | 91.47 | 313.64 |
| **Intervention** |  |  |  |  |
| ***Program costs*** |  |  |  |  |
| Capital costs |  | 13,829,238 | 12,430,257 | 15,554,539 |
| Recurrent costs  (per patient in average) |  | 285.09 | 196.61 | 440.17 |
| ***Patient costs*** |  | 22,694.00 | 18,861 | 29,142 |
